# G. E. Ewbank

**Published:** 1825-06

**Authors:** 


### OBITUARY.

Died, on Monday, the 16th instant, at his house in Lower Brook-street, G. E. Ewbank, Esq. aged thirty-six, lately one of the surgeons to St. George's Hospital. Mr. Ewbank had been suffering, for more than a year, from symptoms, at first denoting great functional derangement of the digestive organs, but which latterly had assumed a more formidable aspect, though the precise nature of the disease which destroyed him was not apparent: it is, therefore, much to be regretted that no examination of the body after death was permitted.

In announcing the premature death of this gentleman, we pay but a just tribute to his memory, when we say that his uniform complacency of temper, his good sense, and his unwearied and zealous pursuit of his professional duties, will long live in the memory of his friends.

